# Endometrial Intracrinology: Oestrogens, Androgens and Endometrial Disorders

**DOI:** 10.3390/ijms19103276

**Published:** 2018-10-22

**Authors:** Douglas A. Gibson, Ioannis Simitsidellis, Frances Collins, Philippa T.K. Saunders

**Affiliations:** Centre for Inflammation Research, The University of Edinburgh, EH16 4TJ Edinburgh, UK; d.a.gibson@ed.ac.uk (D.A.G.); ioannis.simitsidellis@ed.ac.uk (I.S.); f.collins@ed.ac.uk (F.C.)

**Keywords:** decidualisation, oestradiol, aromatase, testosterone, dehydroepiandrosterone (DHEA), endometriosis, endometrial cancer, sulfatase

## Abstract

Peripheral tissue metabolism of steroids (intracrinology) is now accepted as a key way in which tissues, such as the endometrium, can utilise inactive steroids present in the blood to respond to local physiological demands and ‘fine-tune’ the activation or inhibition of steroid hormone receptor-dependent processes. Expression of enzymes that play a critical role in the activation and inactivation of bioactive oestrogens (E1, E2) and androgens (A4, T, DHT), as well as expression of steroid hormone receptors, has been detected in endometrial tissues and cells recovered during the menstrual cycle. There is robust evidence that increased expression of aromatase is important for creating a local microenvironment that can support a pregnancy. Measurement of intra-tissue concentrations of steroids using liquid chromatography–tandem mass spectrometry has been important in advancing our understanding of a role for androgens in the endometrium, acting both as active ligands for the androgen receptor and as substrates for oestrogen biosynthesis. The emergence of intracrinology, associated with disordered expression of key enzymes such as aromatase, in the aetiology of common women’s health disorders such as endometriosis and endometrial cancer has prompted renewed interest in the development of drugs targeting these pathways, opening up new opportunities for targeted therapies and precision medicine.

## 1. What Do We Mean by ‘Intracrinology’?

The term ‘intracrine’ emerged in the 1980s as a new concept in endocrinology based on the ability of cells within non-gonadal tissues to both produce (synthesise) a hormone (peptide, protein or steroid) and to respond to that same factor [1,2]. For many researchers working in the field of sex steroid hormones, the ‘At the cutting edge’ review by Fernand Labrie published in 1991 and simply titled ‘Intracrinology’ was the paper that first made them expand their horizons beyond thinking of gonad-derived sex steroids as the only regulators of steroid target tissues such as the endometrium [1]. That review made what at the time appeared to be a bold claim, that the ‘best estimate of the intracrine formation of oestrogens in peripheral tissues in women is in the order of 75% before menopause, and close to 100% after menopause’. In recent years, particularly following increasing use of liquid chromatography–tandem mass spectrometry (LC-MS/MS), there has been an increase in the number of studies providing evidence for changes in tissue-specific concentrations of steroids that did not necessarily parallel those in blood, as well as increased understanding in the range and number of enzymes and pathways under consideration [3,4,5,6]. Peripheral tissue metabolism of steroids is now accepted as a key way in which tissues such as the endometrium can respond to local physiological demands and ‘fine-tune’ the activation or inhibition of steroid hormone receptor-dependent processes. The original concept of ‘intracrine’ regulation was defined as involving both biosynthesis and response within the same cell (see Figure 1 in [1]) to distinguish it from autocrine or paracrine regulation. However, in more recent studies and reviews, ‘intracrinology’ is now usually discussed on the basis that it is tissue-specific, local production (and inactivation) of sex steroids without significant release of active sex steroids into the peripheral circulation [1,5,6], with less attention being paid to the source and site of action being in the same cell and a greater emphasis on the tissue micro-environment. In this regard, a strong case has been made that the ‘inactive’ adrenal steroid dehydroepiandrosterone (DHEA) is the most important precursor of bioactive androgens in women [7]. There has also been a rapid increase in the number of studies considering the role of locally produced (intracrine) steroids in the aetiology of pathologies including cancers of the breast [8,9] and endometrium [10,11,12], as well as in regulation of fertility [13] and the aetiology of the oestrogen-dependent disorder endometriosis [14]. In the current review, we have based our discussion on the evidence for ‘local’ production and/or activation of steroids that may act in an intracrine, autocrine or paracrine manner within the endometrium or associated disorders.

In the following sections we will provide a brief overview of the structure of the endometrium, its regulation by ovarian-derived steroids and expression of steroid receptors as the prelude for a review of the evidence supporting a role for local tissue activation/biosynthesis of bioactive oestrogens (oestrone, E1: oestradiol, E2) and androgens (testosterone, T: dihydrotestosterone, DHT) in the normal endometrium and in some disorders associated with endometrial malfunction. We will also briefly review the evidence that supplementation with inactive steroids or administration of drugs targeting intracrine steroid biosynthesis may offer a new therapeutic opportunity to treat a range of disorders, including infertility.

## 2. Endometrium—A Sex Hormone-Dependent Multicellular Tissue

### 2.1. Endometrial Tissue Structure and Response to Ovarian-Derived Hormones

**Figure 1:**
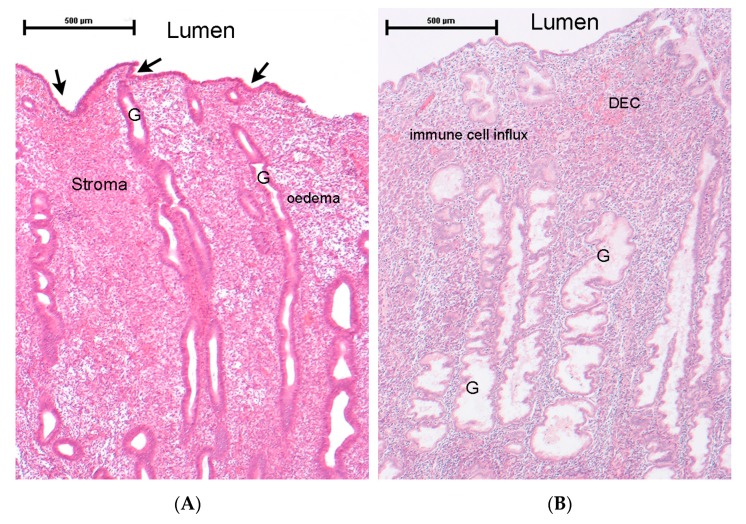
Histology of the human endometrium during the normal cycle. Full-thickness endometrial biopsies from hysterectomy specimens stained with haematoxylin and eosin. (**A**) Proliferative phase: note the presence of long curving glands (G) and some stromal oedema [23]. (**B**) Secretory phase: note the prominent glands (G), which have a dilated lumen and an irregular outer border stretching down into the basal compartment [24]. In the luminal (functional layer) immune cells are readily detected (most of these are likely to be macrophages and uterine natural killer (uNK) cells [17,19]), as are areas of decidualised fibroblasts (DEC) close to arterioles.

The human endometrium is located within the central area of the uterus, surrounded by the muscular layers of myometrium, with a layer of epithelial cells providing an interface between the tissue and the luminal compartment (Figure 1) [15]. Histologically, the human endometrium has two distinct layers: an outer basal compartment and an inner functional compartment. Both layers contain glands bounded by epithelial cells embedded in a multicellular stroma consisting of fibroblasts, resident immune cells and an extensive vascular compartment (endothelial cells, pericytes and vascular smooth muscle). In response to fluctuating changes in the concentrations of oestrogen and progesterone circulating in the blood as a result of changes in ovarian function, the tissue also experiences cyclical episodes of proliferation (oestrogen-dominated proliferative phase), differentiation of stromal fibroblasts (progesterone-dominated secretory phase), and in the absence of a pregnancy, breakdown of the inner (functional) layer, shedding and scarless healing (menstruation).

We, and others, have shown that changes in endometrial tissue function are characterised by changes in the architecture of the glands and differentiation of stromal cells (decidualisation) (Figure 1B) [16]. These are accompanied by changes in both the number and population of resident immune cells that play key roles in regulating differentiation of the tissue vasculature in preparation for implantation and in endometrial tissue repair at the time of menstruation [17,18,19,20]. It is notable that many authors have classified menstruation as an ‘inflammatory event’ highlighting increased concentrations of prostaglandins, which may also be generated by intracrine mechanisms, involving local enzyme expression, as well as increased synthesis of pro-inflammatory cytokines, chemokines and matrix metalloproteinases [21,22].

### 2.2. Expression of Androgen and Oestrogen Receptors in the Endometrium, Endometriosis and Endometrial Cancer

Steroid hormone action is classically mediated by intracellular proteins encoded by members of the nuclear receptor subfamily NR3: there is a single androgen receptor gene *NR3C4* (*AR*) located on the X-chromosome and two oestrogen receptor genes *NR3A1* (*ESR1*, oestrogen receptor alpha) and *NR3A2* (*ESR2*, oestrogen receptor beta) located on chromosome 6 and chromosome 14, respectively, in women (https://www.nursa.org/nursa/index.jsf). These receptors may act within the nucleus as ligand-activated transcription factors by several different mechanisms: (1) binding directly to hormone ‘responsive elements’ on target genes for example DNA sequences shown to have specificity for the androgen receptor (androgen response element, ARE) or either of the oestrogen receptors (estrogen response element, ERE); (2) in association with the binding of other transcription factors (AP-1, Sp-1) or (3) acting outside the nucleus via rapid, ‘non-genomic’ pathways—the evidence for all of these pathways has recently been extensively reviewed and will not be discussed further [25,26]. Detailed immunohistochemical studies using fixed full-thickness endometrial tissues in combination with validated antibodies [27] have identified nuclear staining for AR, ERalpha and ERbeta in the human endometrium during the normal menstrual cycle [16,18,28,29]. Notably, the tight spatial and temporal localisation of these receptors can provide insights into the cells that may be influenced by the actions of intracrine-derived steroids. Notably, key target cells for androgens are endometrial fibroblasts, which are AR-positive in the functional layer during the proliferative phase and in the basal compartment throughout the cycle. AR is downregulated in stromal cells in the functional layer during the secretory phase and upregulated in epithelial cells when progesterone levels decline (functional withdrawal with demise of the corpus luteum) [28] or in response to administration of progesterone receptor antagonists/selective modulators [30].

In the human endometrium, ERalpha and ERbeta exhibit cell-specific patterns of expression during the menstrual cycle [29]. Notably, ERalpha is present in the epithelial cells lining the glands and lumen during the proliferative phase, at a time when circulating oestrogens are rising rapidly due to growth of antral follicles containing granulosa cells expressing aromatase [31], but is downregulated during the secretory phase [29]. Immunolocalisation of ERbeta protein suggests it does not mirror the dynamic change in expression in stromal or epithelial cells seen with ERalpha and that the protein is present in endothelial cells and multiple populations of immune cells that are ERalpha-negative [17,18,29]. As discussed below, the identification of ERbeta in endothelial and immune cells is consistent with evidence for direct actions of E2 on these cell types. Studies using targeted deletion of *Esr1* and *Esr2* in mice have reported E2-dependent signalling via ERalpha is critically important for stromal-epithelial interactions in the endometrium and epithelial cell proliferation. Uterine epithelial ERalpha is dispensable for proliferation but essential for complete biological and biochemical responses; [32]. Global ablation of *Esr2* resulted in a predominant ovarian phenotype [33]. There is also evidence for the expression of variant isoforms of both subtypes in the human endometrium formed by translation of mRNAs generated by alternative splicing of the *ESR1* and *ESR2* genes [25,29,34]: these variants are not present in mice and their function is poorly understood.

Comparison between the patterns of expression of receptor proteins in normal endometrium with samples of endometrial cancer and endometriosis lesions has revealed evidence of aberrant expression of both AR and ERs, which may result in novel disease-specific targets for the action of steroids generated by intracrine activation/metabolism discussed below. Examples include epithelial cell expression of AR in endometrial cancers [26] and upregulation of ERbeta in endometriosis [35].

## 3. Methodology

On 4 May 2018 searches were conducted of the PUBMED (https://www.ncbi.nlm.nih.gov/pubmed/) and SCOPUS (https://www.scopus.com/search/form.uri?display=basic) databases using a range of terms. A variable number of references, some of which overlapped, were identified: ‘intracrinology’ (167 references Scopus); intracrinology[and]endometrium (11 references Pubmed/21 references Scopus). These basic searches were expanded by considering individual enzymes known to be implicated in intracrine biosynthesis and pathologies: this yielded a larger number of references, probably indicating that local expression of enzymes in tissues is not always tagged as being indicative of ‘intracrinology’. Examples from Pubmed searches include aromatase[and]endometrium = 398, aromatase[and]endometrium[and]cancer[and human] = 135; sulfatase[and]endometrium = 84: HSD[and]endometrium = 131.

## 4. Intracrine Steroid Biosynthesis in the Normal Endometrium

Classically, the endometrium was considered a target for endocrine hormones, with early studies focusing on the capacity for the tissue to metabolise (inactivate) steroids. Subsequent more detailed investigations have demonstrated that the endometrium expresses enzymes capable of biosynthesis, as well as metabolism of steroids, with endometrial cells having the capacity to enzymatically convert androgens into oestrogens, as well as to utilise adrenal and sulphated steroid precursors [36]. Notably, the capacity to convert different substrates was found to vary with menstrual cycle phase, characterised by increased conversion of DHEA, and formation of T during the secretory phase [36]. Although biosynthesis of active steroids was known to be a feature of endometrial disorders such as endometriosis and endometrial cancer (see Section 5), it has only become apparent in the last 10 years that intracrine steroid biosynthesis and metabolism plays an important role in the regulation of normal endometrial function and fertility.

### 4.1. Insights Gained from Measurement of Steroid Precursors and Metabolites in Endometrial Tissue

Aided by improvements in sensitivity of LC/MS-MS for measurement of oestrogens, the Poutanen group and their collaborators compared concentrations of E1 and E2 in blood and in matched endometrial tissue biopsies from individual women. Strikingly, they found that concentrations of oestrogens were higher in endometrial tissue than in the circulation and that concentrations of E2 were increased in secretory phase compared to proliferative phase tissues [3,37], with the latter finding being consistent with increased expression of enzymes such as aromatase or 17β-HSD in response to a decidualisation stimulus (see Section 4.3). In complementary studies, the concentrations of androgens and progestins were also measured in blood and in matched endometrial tissue. Notably, intra-tissue concentrations of the androgen precursor DHEA were significantly increased compared to serum concentrations in samples collected from the secretory phase. In contrast, they found that concentrations of androstenedione (A4) and T were significantly lower in endometrial tissue homogenates than in the serum and that these were not cycle phase-dependent [3].

### 4.2. Androgen Activation and Metabolism

Androgen biosynthesis within tissues can arise from de novo steroidogenesis, such as in the ovary, or via conversion of androgen precursors in extragonadal tissues. De novo steroid biosynthesis requires cholesterol, which is shuttled to the inner mitochondrial membrane by steroidogenic acute regulatory protein (StAR), where it undergoes side-chain cleavage by CYP11A1 (Cholesterol side chain-cleavage enzyme) to yield pregnenolone (P5). P5 undergoes two enzymatic conversions mediated via CYP17A1, first to 17α-hydroxypregnenolone (17α-hydroxylase activity) and then following 17,20 lyase action to yield DHEA [38]. DHEA is produced by the adrenal glands and by the ovary and is abundant in the circulation. Expression of StAR, CYP11A1 and CYP17A1 has been reported in the endometrium [39]; however, to the best of our knowledge no study has identified definitive de novo steroidogenesis from normal endometrial tissue. 

**Figure 2:**
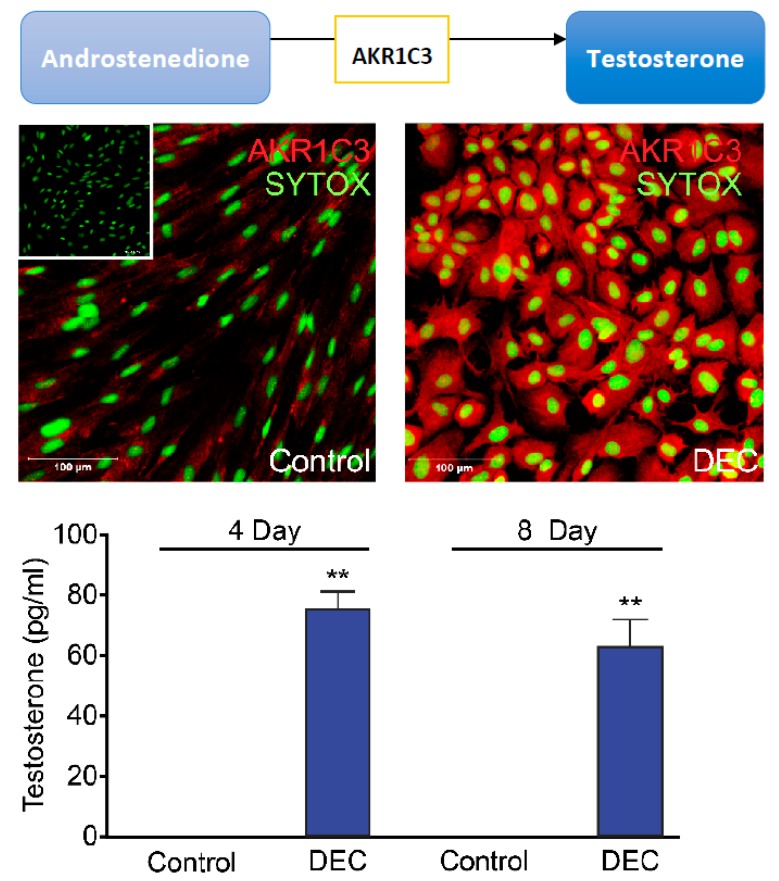
Expression of AKR1C3 in human endometrial stromal fibroblasts is increased in response to a decidualisation stimulus resulting in increased biosynthesis and secretion of testosterone. Based on a figure published in [43] under a CC-BY licence: concentrations of T were determined using an ELISA on days 4 or 8 of the experiment. Immunohistochemistry was determined on day 4 of experiment; AKR1C3 (red stain) Sytox Green nuclear stain (green), no primary antibody negative control (inset) (** *p* < 0.01).

DHEA and its sulphated form dehydroepiandrosterone sulphate (DHEAS) are abundant in the circulation and can be utilised as precursors by endometrial cells. We and others have reported that 3β-hydroxysteroid dehydrogenase (3βHSD) is expressed in endometrial stromal cells [13] and that during decidualisation DHEA can be utilised as an androgen precursor yielding both A4 and T via 3βHSD [3,40,41]. Following conversion of DHEA to A4 by 3βHSD, activation of androgen agonists T and DHT is tightly controlled via the action of aldo-keto reductase family 1 member C3 (AKR1C3). Assessment of endometrial tissue samples by immunohistochemistry and qRT-PCR of whole-tissue homogenates performed by Catalano et al. demonstrated that AKR1C3 expression is increased in the secretory phase relative to proliferative phase with peak expression reported in the early- to mid-secretory phase [42]. Our studies using an in vitro time course of decidualisation paralleled this expression pattern with peak expression detected between day 2 and 4 of an eight-day decidualisation protocol [43] (Figure 2). In contrast to AKR1C3, expression of 5α-reductase (SRD5A1), which converts T to the more potent and non-aromatisable androgen DHT, is decreased in endometrial cells as decidualisation progresses [43]. Thus, time-dependent conversion of A4 to T determines substrate availability and hence production of DHT.

Interconversion of active/inactive androgens is mediated via 17β-hydroxysteroid dehydrogenase isozymes. Expression of several 17β-hydroxysteroid dehydrogenase (HSD17B) isozymes have been reported in endometrial tissues/cells [44]. In addition to AKR1C3 (also known as HSD17B5), HSD17B7 and 12 are reported to have reductive 17β-HSD activity, mediating conversion of A4 to T, and mRNA expression of *HSD17B7* and *HSD17B12* has been reported in total tissue homogenates of normal human endometrium but with no significant cycle-dependent change in expression [4]. The predominant isoform with oxidative 17β-HSD activity in the endometrium is HSD17B2, which converts active T to A4. Expression of HSD17B2 is increased by progesterone and elevated in the secretory phase [4,45]. HSD17B14 also has oxidative 17β-HSD activity and has been immunolocalised to endometrial glandular epithelial cells [46] although relative efficiency for oxidation of E2 or T was much lower than that of HSD17B2 in cell metabolism assays. Although HSD17B2 has oxidative action on androgens and oestrogens, our own metabolism studies suggest that this activity is decreased in endometrial stromal cells during decidualisation, even when *HSD17B2* mRNA expression is increased [13,43]. This may reflect alternative activity of HSD17B2 during the secretory phase, such as by activating 20α-hydroxyprogesterone to increase bioavailability of P4 [47].

### 4.3. Oestrogen Biosynthesis and Metabolism

There are two main pathways by which oestrogens are synthesised within endometrial cells: (1) conversion of androgens such as A4 and T to active oestrogens, E1 and E2, respectively, via the action of the aromatase enzyme complex, the key component of which is the aromatase protein encoded by the *CYP19A1* gene; (2) conversion of sulphated oestrogens oestradiol sulphate (E2S) or oestrone sulphate (E1S) into their bioactive metabolites (E2 and E1, respectively) via the action of steroid sulphatase encoded by *STS* (discussed below) [13,48].

**Figure 3:**
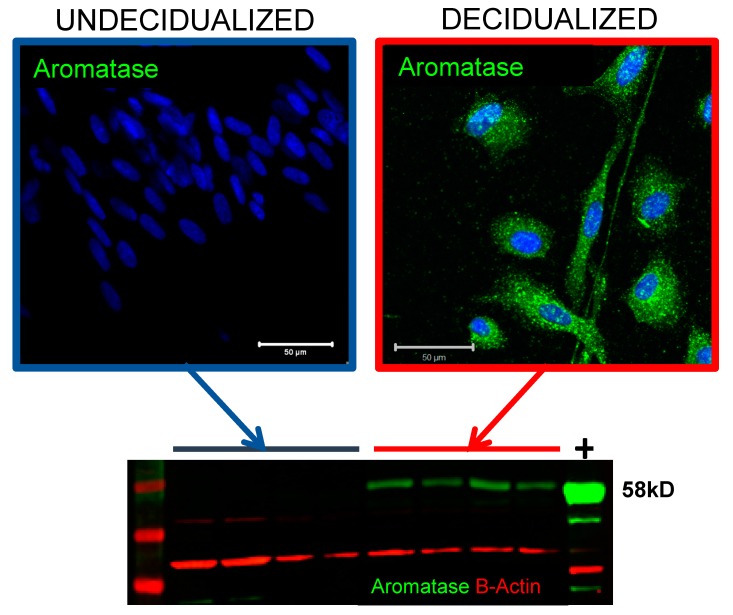
In vitro decidualisation of endometrial stromal fibroblasts results in upregulation of aromatase protein expression (green). Based on data reported in [13]. ESC—primary endometrial stromal fibroblasts; positive control (+) was a protein extract from human placenta, aromatase protein 58kDa. Nuclear counterstain DAPI (blue); Aromatase (green); B-Actin loading control (red).

Landmark studies in mice by the Bagchi group demonstrated that aromatase activity within the endometrium was essential for establishment of pregnancy in that species [49,50]. In a number of elegant experiments they showed that decidualisation and vascular remodelling were impaired if an aromatase inhibitor was administered in vivo [49]. In women, when assessed as whole tissue homogenates, expression of aromatase is low/undetectable in endometrial samples recovered during the normal cycle. However, using primary human endometrial stromal cells we discovered that aromatase protein and enzyme activity were increased following decidualisation resulting in increased synthesis of E1 and E2 [13] (Figure 3). Critically, these studies demonstrated the temporal regulation of this process, whereby expression of aromatase and secretion of E2 increased in a time-dependent manner, while metabolism (inactivation) of E2 to E1 decreased [13], resulting in an increased E2:E1 ratio. We speculate that the failure to detect altered expression in tissue homogenates may reflect tight temporal and/or spatial regulation, which will be missed unless samples are recovered from areas of active decidualisation in the functional layer of the tissue.

Expression of oxidative and reductive 17BHSD isoforms may also contribute to generation of E2 from E1: although both E1 and E2 can activate ERs, E2 is generally considered to be a more potent agonist. Activation of E1 to E2 via reductive 17β-HSD activity is primarily mediated by HSD17B1 but also HSD17B4 and 7 in the uterus. Expression of these enzymes has been reported in normal endometrium [4], and as elevated in endometrial disorders (see Section 5). 

### 4.4. Role of Sulphated Steroids as a Source of Endometrial Androgens and Oestrogens

Androgens and oestrogens can be metabolised from the common precursor DHEAS via the action of steroid sulfatase (STS), which de-sulphates DHEAS to DHEA. Additionally, STS can metabolise E1S and E2S sulphate to bioactive E1 and E2 respectively. STS is expressed in normal endometrial tissues and in endometrial cancers [51,52]. STS expression and activity is increased during decidualisation of endometrial stromal cells in vitro, consistent with a detectable increase in oestrogens detected in tissue samples recovered during the secretory phase [3,48].

Sulfation is a key mechanism for deactivating steroids and is essential for appropriate intracrine regulation within the tissue environment. Sulfation is mediated by sulfotransferase enzymes and requires the action of 3′-phospho-adenosine-5′-phosphosulphate synthase (PAPSS) enzymes to provide a sulphate moiety for conjugation [53]. There is little evidence for expression of DHEA sulphating enzymes/activity in the endometrium, although expression of *SULT1A1* and *SULT2B1a* were reported in endometrial stromal cells together with *PAPSS1* and *PAPSS2* [48]. The main oestrogen sulphotransferase (SULT1E1) is expressed in endometrial epithelial cells and expression is increased in response to progesterone [54] highlighting a potentially complex balance between activation and inactivation of steroids during the fertile phase of the cycle that may involve more than one cell type.

### 4.5. Intracrine and Paracrine Impact of Tissue Biosynthesis of Androgens and Oestrogens in Endometrium

Perivascular decidualisation is associated with accumulation of specialised immune cells known as uterine natural killer cells (uNK) that are critical mediators of vascular remodelling in early pregnancy. In common with other immune cells found in the endometrium (macrophages, mast cells [55,56]), uNK cells express ERbeta [18]. Strikingly, we have demonstrated that conditioned media from decidualised stromal cells increases uNK migration in an oestrogen-dependent manner. In addition, treatment of uNK cells with E2 resulted in increased secretion of angiogenic factors, including CCL2, which had a significant impact on endometrial endothelial cell network formation [57]. The impact of a local oestrogen-rich microenvironment on the function of endometrial macrophages or mast cells has yet to be elucidated. Recent evidence suggests that sulphated oestrogen precursors are also utilised by endometrial stromal cells as intracrine/paracrine regulators, as secretion of E1 and IGFBP1, a prominent decidualisation marker, are decreased in the presence of the STS inhibitor STX64 (Irosustat) [48]. Other studies that have highlighted oestrogen-dependent changes in endometrial endothelial cell gene expression [58] that will also be influenced by local (intracrine) metabolism/biosynthesis of bioactive oestrogens. In summary, the evidence presented in the studies described above, together with those in mice [49], all support the hypothesis that tight temporal-spatial regulation of tissue function by intracrine oestrogens plays a previously underappreciated role in modulating the function of cells (endothelial, uNK, decidual fibroblasts) that play key role(s) in the formation of a receptive endometrium capable of supporting a viable pregnancy.

Endometrial biosynthesis of androgens has also emerged as a regulator of fertility and endometrial function. Specifically, human primary stromal cells are AR-positive and treatment of cells from proliferative phase endometrium with DHT has identified a number of androgen-regulated genes [28]. Complementary studies by Gibson et al. [43], in which primary cells were stimulated to decidualise, showed local intracrine biosynthesis of androgens may play a critical role in maintenance of a decidual phenotype, with addition of flutamide reducing biosynthesis of the decidualisation marker IGFBP1 [43,59]. These studies complement and extend those of Cloke et al. [60,61], who showed that AR and PR regulated distinct genomic pathways during decidualisation, consistent with a role for androgens (either local or peripheral) in regulation of fertility. Studies in mice have also identified a potential role for androgens in endometrial repair at the time of menstruation [62] at a time when peripheral oestrogens are low, which merits further investigation. 

The identification of aromatase expression and production of oestrogens in both mouse tissue and human endometrial cells has highlighted the importance of locally produced androgens acting as precursor steroids for oestrogen biosynthesis. Results from studies reporting that intra-tissue concentrations of androgens T and A4 were lower than serum [3] would be consistent with this hypothesis. However, one caveat is that these studies assessed whole endometrial tissue homogenates, which do not allow for the contribution of specific cellular compartments to be quantified. We therefore investigated the temporal dynamics of androgen metabolism using an in vitro model of isolated primary human endometrial stromal cells. In these studies, treatment of cells with the AR antagonist flutamide, reduced secretion of both decidualisation and endometrial receptivity markers [43]. We further demonstrated that supplementation with the androgen precursor DHEA increases biosynthesis of T and DHT and is associated with dose-dependent increases in expression of the decidualisation markers IGFBP1 and prolactin, as well as the endometrial receptivity marker SPP1 [40]. Taken together, these studies suggest both local activation and metabolism of androgens occur during decidualisation and that temporal regulation of intracrine androgen bioavailability is a critical mediator of endometrial competence during remodelling required for establishment of pregnancy [39,59]. These studies are of particular relevance to the impact of aging on fertility, as circulating concentrations of androgens precursors, such as DHEA and A4 as well as T and DHT decline with age [38,63].

## 5. Evidence for the Importance of Intracrinology in Endometrial Disorders

### 5.1. Endometriosis

Endometriosis is a chronic oestrogen-dependent disorder that is characterised by growth of endometrial cells/tissue fragments (lesions) outside the uterus [64]: there are three broad classifications based on location of the lesions, peritoneal, ovarian (endometriomata) and deep infiltrating lesions in the rectovaginal area. A recent review provides an excellent overview of the features of endometriosis as determined by magnetic resonance imaging [65]. Common histologic features of all three manifestations include the presence of endometrial-like cells (either stromal and/or glandular, vascular cells and nerves) as well as evidence of inflammation (immune cell populations). Endometriosis can be associated with debilitating pelvic pain and in most but not all women, symptoms regress after menopause, with many of the drug regimens used to treat symptomatic endometriosis being based on suppression of ovarian cyclicity [64]. Examination of ectopic lesions revealed that they are characterised by high aromatase expression levels, together with a deficiency in 17β-HSD2, the enzyme responsible for the inactivation of E2 to E1 [66]. A recent Cochrane review concluded that, ‘for women with pain and endometriosis, suppression of menstrual cycles with gonadotrophin-releasing hormone (GnRH) analogues, the levonorgestrel-releasing intrauterine system (LNG-IUD) or Danazol were beneficial interventions’ [67]. However, it is notable that for many women, particularly those wishing to conceive, ovarian suppression is not desirable as the associated menopausal side effects can be severe and intracrine mechanisms are not suppressed. Whilst our literature search terms ‘intracrine and endometriosis’ only identified five papers on PUBMED it is notable that the alternative search ‘aromatase and endometriosis’ identified more than 300, reflecting widespread interest in the role of local (intracrine) biosynthesis in the aetiology of this complex disorder and the potential that this might provide a novel therapeutic opportunity [68]. 

Local oestrogen production, accompanied by intracrine and paracrine signalling via ERbeta in endometriotic tissues is believed to contribute to a feed-forward signalling cascade that maintains an inflammatory state and cell proliferation within the endometriotic lesions (for a comprehensive review on the role of oestrogen production and action in endometriosis see [69]). Peritoneal fluid from women with endometriosis contains high concentrations of pro-inflammatory cytokines, such as TNFα and IL-1β [70,71], which have the capacity to stimulate expression of the prostaglandin synthesis enzyme COX-2 and increase secretion of prostaglandin E2 (PGE_2_) by endometriotic cells and peritoneal macrophages [72,73]. PGE_2_ in turn stimulates the production of cyclic AMP (cAMP), which, together with steroidogenic factor 1 (SF1), whose expression is aberrantly upregulated in endometriotic tissue compared to endometrial tissue, induces expression of mRNAs that encode enzymes that play a critical role in the steroidogenic enzyme machinery, including STAR, 17-hydroxylase/17,20-lyase, 3β-HSD and aromatase, which may be consistent with the synthesis of E2 from cholesterol within lesions [72,74]. 

The significance of intracrine signalling in the aetiology of endometriosis has been supported by Huhtinen et al., who used LC/MS-MS to interrogate the concentrations of steroids in matched endometrial, endometriotic (lesions) and serum samples from women with or without endometriosis [3,4]. A striking finding from these studies was that the concentrations of testosterone in endometriotic lesions (both ovarian and extra-ovarian) far exceeded those in the blood regardless of menstrual cycle stage [3]. This increase was mirrored by elevated expression of *CYP11A1*, *CYP17A1* and *HSD3B2* in endometriotic lesions, especially those associated with the ovary (endometriomas), compared to intrauterine endometrium. It was also accompanied by significant changes in expression of androgen-regulated genes (*PRUNE2*, *HGD*, *PDGFRL*) [3] providing a readout of the action of androgens binding to AR expressed in the lesions. Thus, increased intra-tissue testosterone synthesis in endometriotic lesions, as well as providing a substrate for aromatase and biosynthesis of E2, may promote the activation of an AR transcriptional network within the lesions. 

Apart from the discrepancy between intra-tissue steroid concentrations and those in the circulation, there are also significant variations in the levels of endometrial and circulating steroid hormones that are menstrual cycle-dependent. Using the same experimental approach described above, Huhtinen et al. reported that while endometrial and endometriotic intra-tissue concentrations of E2 were significantly higher compared to those in the serum of women in the proliferative phase of the menstrual cycle, the opposite is the case for the secretory phase [4]. Moreover, expression of *HSD17B2* was significantly lower within lesions compared to endometrial tissue while expression of *HSD17B6* and *CYP19A1* was significantly higher [4]. It must be noted that there is a distinct difference both in the local steroid concentrations and the expression of steroid metabolising enzymes within different types of lesions. For example, in the proliferative phase, E2 concentrations in ovarian lesions (endometrioma) were approximately 3430 pg/mL, while in peritoneal lesions, E2 concentrations were 238 pg/mL [4]. This demonstrates a heterogeneity in intracrine steroid action within different types of lesions, which in the case of ovarian lesions could derive from the proximity of the endometriotic cells to ovarian follicles and the constant supply of steroids from the follicles within the ovaries. Further studies are required to explore this possibility.

### 5.2. Endometrial Cancer

Endometrial cancer (EC) is the fourth most common cancer in women, with the majority of women being diagnosed at early stages of the disease following a uterine bleed after menopause [cancerresearchuk.org]. Established risk factors for development of EC include obesity and the presence of premalignant lesions associated with endometrial hyperplasia [75,76], with oestrogen exposure considered a key driver of both endometrial hyperplasia and Type I EC that make up 75% of EC cases [76]. Rizner and colleagues have recently provided a comprehensive overview of the different studies contributing to our current understanding of the mechanisms that contribute to increased bioavailability of oestrogens in EC [52,77]. A few key studies are described below. 

Expression of the *CYP19A1* gene is regulated by tissue-specific promoters, distributed over a 93kb regulatory region, which have been the subject of extensive investigation [78]. Notably, Bulun and colleagues have found that in cancers of breast, endometrium and ovary, expression is primarily regulated by increased activity of the I.3/II promoter region, which can be upregulated by prostaglandins such as PGE2, providing a link between the overexpression of PGs that has been reported to occur in EC [79] and intracrine oestrogen biosynthesis [80]. Sasano and collaborators have developed novel antibodies and reported evidence of increased immunoexpression of aromatase [81], STS [82] and 17βHSD enzymes [83] in endometrial hyperplasia and EC. These findings are all consistent with increased bioavailability of E1/E2 in association with malignant transformation. Notably, expression of aromatase and 17βHSD 1 in EC have both been correlated with poor prognosis [84,85].

We and others have shown that AR are widely expressed in EC and also in EC cell lines such as Ishikawa, which have been extensively studied (reviewed in [26]). In a recent study, Kamal et al. [86] reported that the expression of AR was downregulated in high-grade EC but elevated in metastases, raising the possibility that they might be a target for therapy. In a recent review, Ito et al. summarised the epidemiological data supporting an association between elevated androgens in the circulation and the risk of developing EC [11]. Whilst there has been less interest in the intracrine generation of androgens other than as substrates for aromatase, it is notable that a study by the same group revealed that EC tissues had an 8-fold elevation in DHT compared with that in normal endometrial tissues [87]. The same study compared AR expression with that of 5 alpha reductase enzymes 1 and 2 (5αR1 and 5αR2), concluding that expression of 5αR1 (65% of samples) was positively correlated with histological grade (but not clinical grade). They found that women immunonegative for both AR and 5αR1 had a poorer prognosis [87], consistent with other studies that have suggested androgens can be anti-proliferative for EC cells.

## 6. Intracrinology and Metabolism

Whilst this review has focused on endometrial tissue and its pathologies, there is a growing body of evidence showing the importance of intracrine metabolism in non-reproductive tissues. For example, in their recent comprehensive review of intracrine androgen biosynthesis and metabolism, Schiffer and colleagues highlighted the importance of peripheral metabolism of steroids in metabolic target tissues including adipose and skeletal muscle [88]. Studies on the role of AR in skeletal muscle conducted using transgenic mouse models have shown it is expressed in multiple cell types in muscle [89,90]. The pharmaceutical industries have developed a number of selective androgen receptor modulators to target AR in muscle as a therapy for age-related or cancer-related loss of muscle function [91,92]. 

## 7. New Therapeutic Approaches for Treatment of Endometrial Disorders Based on Intracrine Targets

Labrie and colleagues have conducted several studies and clinical trials to analyse the impact of intravaginal administration of DHEA (Prasterone; brand name in USA Intrarosa) on adverse symptoms resulting from postmenopausal steroid deprivation including vaginal atrophy and pain during sexual intercourse [93]. They have demonstrated positive impacts on vaginal dryness and other steroid hormone-dependent parameters without any evidence of peripheral changes in serum E2 [94,95]. In other studies, significant benefit for vaginal health has been demonstrated using estriol gel [96]: these data have collectively provided powerful evidence for intracrine steroid modulation playing a key role in regulation of the vaginal microenvironment, which can be harnessed for therapeutic benefit after ovarian secretion of oestrogens stops at menopause [97]. A recent study reported supplementation of culture media with DHEA enhanced expression of receptivity genes by decidualised stromal cells from women with a mean age of 44. Thus there is the potential that administration of DHEA during the secretory phase alone may also assist fertility in older women, although this clearly requires further evaluation [40].

In other studies the emphasis has been on inhibition of enzymes that appear dysregulated in disease. For example, there are a number of reports that aromatase is expressed in endometriosis lesions with evidence of a positive feed-forward loop involving local biosynthesis of both E2 and the pro-inflammatory regulator prostaglandin E2 [69,98]. These have been complemented by several studies reporting on the positive impact, or lack thereof, of aromatase inhibitors (AI) including letrozole and anastrozole on symptoms of endometriosis in both pre- and post-menopausal women [99,100]. The current consensus is that AI should be considered as a treatment for endometriosis-associated pain in women who are postmenopausal but still symptomatic as it will target intracrine oestrogen biosynthesis, which is thought to play an important role in this age group [99].

To target intracrine biosynthesis of steroids and prostaglandins, Bayer Pharma has developed an AKR1C3 inhibitor as a therapy for endometriosis (BAY 1128688). A phase I trial was completed in 2016 and a phase II trial is listed as underway in Spain (EudraCT number 2017-000244-18) with results awaited as to efficacy. Insulin stimulates AKR1C3 expression in the adipose tissue of women with PCOS and its inhibition has been suggested as offering a therapeutic target to reduce the hyperandrogenism that is a feature of this disorder [101].

A number of 17βHSD inhibitors have been developed to target intracrine biosynthesis of oestrogens in hormone-dependent disorders, including cancer and endometriosis [102]. Promising results have been reported using a 17βHSD1 inhibitor to suppress conversion of E1 to E2 in endometriosis tissue homogenates [103]. Forendo Pharma, based in Turku, Finland (http://www.finlandhealth.fi/-/forendo-pharma), has developed a specific 17βHSD1 inhibitor (FOR-6219) for which a phase Ia trial was initiated in July 2018. High expression of 17βHSD1 is associated with poor prognosis in EC [84]. In a recent study, Konings et al. [104] reported detection of 17βHSD1 in EC metastatic lesions and had promising results demonstrating the inhibition of enzyme activity using the FP4643 type 1 inhibitor in both in vitro and ex vivo models.

The evidence that STS is expressed in endometrial cancers [52,105] and endometriosis [106] has prompted the development of specific STS inhibitors as novel therapies. Several potent STS inhibitors have been developed [107], including STX64, which was effective in blocking synthesis in endometrial cancer cells in vitro. STX64 has been renamed Irosustat (Ipsen) and was tested in a phase 2 open-label trial in women with advanced/metastatic or recurrent oestrogen receptor-positive endometrial cancer, but did not result in better survival rates and so has not been developed further. 

Another inhibitor, estradiol-3-*O*-sufamate (E2MATE), used on human endometrial explants and in a mouse model of endometriosis, has shown some promising results [108]. Currently under the name PGL2, this compound is listed on the web as being part of a phase II clinical trial for endometriosis (Jenapharm: https://adisinsight.springer.com/drugs/800026648). 

Whilst monotherapies are still under testing, drugs that have dual action have also been developed to target both aromatase and STS (DASI), as well as STS and 17βHSD1. A dual STS/17βHSD1 inhibitor has been shown to block the proliferation of cancer cells treated with E1S/E1 but not those treated with E2 alone [109], but this awaits further testing in vivo. A range of DASI drugs have been developed and tested by Barry Potter and his colleagues, with promising results in cell and animal models (reviewed in [110]), but have yet to be tested as treatment for women with EC or endometriosis. 

## 8. Conclusions

Intracrine regulation of oestrogens and androgens has emerged as a key regulator of endometrial function, both during the normal cycle and in endometrial disorders such as cancer and endometriosis. Further studies are needed to better define their role in the complex cross-talk between different cell types and the interplay between metabolic and inflammatory processes. To date, regulation of normal and abnormal endometrial tissue resulting from intracrine biosynthesis of steroids has focused on regulation of gene expression; however, with new evidence that a wide range of non-coding RNAs are also likely to play a role in endometrial tissues, studies need to be devised to explore whether their availability is also regulated by steroids [111]. Importantly, the manipulation of intracrine sex steroid metabolism has emerged as a therapeutic target for the treatment of endometriosis and endometrial cancer and the scope of these studies is likely to broaden as we gain a greater understanding of their roles in fertility.

## References

[1] Labrie F. (1991). Intracrinology. Mol. Cell. Endocrinol..

[2] Re R.N. (1989). The cellular biology of angiotensin: Paracrine, autocrine and intracrine actions in cardiovascular tissues. J. Mol. Cell. Cardiol..

[3] Huhtinen K., Saloniemi-Heinonen T., Keski-Rahkonen P., Desai R., Laajala D., Stahle M., Hakkinen M.R., Awosanya M., Suvitie P., Kujari H. (2014). Intra-tissue steroid profiling indicates differential progesterone and testosterone metabolism in the endometrium and endometriosis lesions. J. Clin. Endocrinol. Metab..

[4] Huhtinen K., Stahle M., Perheentupa A., Poutanen M. (2012). Estrogen biosynthesis and signaling in endometriosis. Mol. Cell. Endocrinol..

[5] Labrie F., Luu-The V., Labrie C., Simard J. (2001). DHEA and its transformation into androgens and estrogens in peripheral target tissues: Intracrinology. Front. Neuroendocrinol..

[6] Labrie F., Luu-The V., Lin S.X., Simard J., Labrie C., El-Alfy M., Pelletier G., Belanger A. (2000). Intracrinology: Role of the family of 17 beta-hydroxysteroid dehydrogenases in human physiology and disease. J. Mol. Endocrinol..

[7] Labrie F., Martel C., Belanger A., Pelletier G. (2017). Androgens in women are essentially made from DHEA in each peripheral tissue according to intracrinology. J. Steroid Biochem. Mol. Biol..

[8] Suzuki T., Moriya T., Ishida T., Ohuchi N., Sasano H. (2003). Intracrine mechanism of estrogen synthesis in breast cancer. Biomed. Pharmacother..

[9] McNamara K.M., Sasano H. (2015). The intracrinology of breast cancer. J. Steroid Biochem. Mol. Biol..

[10] Smuc T., Rupreht R., Sinkovec J., Adamski J., Rizner T.L. (2006). Expression analysis of estrogen-metabolizing enzymes in human endometrial cancer. Mol. Cell. Endocrinol..

[11] Ito K., Miki Y., Suzuki T., McNamara K.M., Sasano H. (2016). In situ androgen and estrogen biosynthesis in endometrial cancer: Focus on androgen actions and intratumoral production. Endocr.-Relat. Cancer.

[12] Rizner T.L. (2013). Estrogen biosynthesis, phase I and phase II metabolism, and action in endometrial cancer. Mol. Cell. Endocrinol..

[13] Gibson D.A., McInnes K.J., Critchley H.O., Saunders P.T. (2013). Endometrial Intracrinology—Generation of an estrogen-dominated microenvironment in the secretory phase of women. J. Clin. Endocrinol. Metab..

[14] Rizner T.L. (2009). Estrogen metabolism and action in endometriosis. Mol. Cell. Endocrinol..

[15] DallenbachHellweg G., Schmidt D., Dallenbach F. (2010). Atlas of Endometrial Histopathology.

[16] Critchley H.O., Saunders P.T. (2009). Hormone receptor dynamics in a receptive human endometrium. Reprod. Sci..

[17] Thiruchelvam U., Dransfield I., Saunders P.T., Critchley H.O. (2013). The importance of the macrophage within the human endometrium. J. Leukocyte Biol..

[18] Henderson T.A., Saunders P.T., Moffett-King A., Groome N.P., Critchley H.O. (2003). Steroid receptor expression in uterine natural killer cells. J. Clin. Endocrinol. Metab..

[19] Wilkens J., Male V., Ghazal P., Forster T., Gibson D.A., Williams A.R., Brito-Mutunayagam S.L., Craigon M., Lourenco P., Cameron I.T. (2013). Uterine NK cells regulate endometrial bleeding in women and are suppressed by the progesterone receptor modulator asoprisnil. J. Immunol..

[20] Moffett-King A. (2002). Natural killer cells and pregnancy. Nat. Rev. Immunol..

[21] Evans J., Salamonsen L.A. (2012). Inflammation, leukocytes and menstruation. Rev. Endocr. Metab. Disord..

[22] Maybin J.A., Critchley H.O. (2015). Menstrual physiology: Implications for endometrial pathology and beyond. Hum. Reprod. Update.

[23] Noyes R.W., Hertig A.T., Rock J. (1950). Dating the endometrial biopsy. Fertil. Steril..

[24] Garry R., Hart R., Karthigasu K.A., Burke C. (2011). Structural changes in endometrial basal glands during menstruation. BJOG.

[25] Gibson D.A., Saunders P.T. (2012). Estrogen dependent signaling in reproductive tissues—A role for estrogen receptors and estrogen related receptors. Mol. Cell. Endocrinol..

[26] Gibson D.A., Simitsidellis I., Collins F., Saunders P.T. (2014). Evidence of androgen action in endometrial and ovarian cancers. Endocr. Relat. Cancer.

[27] Saunders P.T.K., Millar M.R., Williams K., Macpherson S., Harkiss D., Anderson R.A., Orr B., Groome N.P., Scobie G., Fraser H.M. (2000). Differential expression of estrogen receptor-α and -β and androgen receptor in the ovaries of marmosets and humans. Biol. Reprod..

[28] Marshall E., Lowrey J., Macpherson S., Maybin J.A., Collins F., Critchley H.O., Saunders P.T. (2011). In silico analysis identifies a novel role for androgens in the regulation of human endometrial apoptosis. J. Clin. Endocrinol. Metab..

[29] Critchley H.O., Brenner R.M., Henderson T.A., Williams K., Nayak N.R., Slayden O.D., Millar M.R., Saunders P.T. (2001). Estrogen receptor β, but not estrogen receptor α, is present in the vascular endothelium of the human and nonhuman primate endometrium. J. Clin. Endocrinol. Metab..

[30] Whitaker L.H., Murray A.A., Matthews R., Shaw G., Williams A.R., Saunders P.T., Critchley H.O. (2017). Selective progesterone receptor modulator (SPRM) ulipristal acetate (UPA) and its effects on the human endometrium. Hum. Reprod..

[31] Turner K.J., Macpherson S., Millar M.R., McNeilly A.S., Williams K., Cranfield M., Groome N.P., Sharpe R.M., Fraser H.M., Saunders P.T. (2002). Development and validation of a new monoclonal antibody to mammalian aromatase. J. Endocrinol..

[32] Winuthayanon W., Lierz S.L., Delarosa K.C., Sampels S.R., Donoghue L.J., Hewitt S.C., Korach K.S. (2017). Juxtacrine Activity of Estrogen Receptor alpha in Uterine Stromal Cells is Necessary for Estrogen-Induced Epithelial Cell Proliferation. Sci. Rep..

[33] Dupont S., Krust A., Gansmuller A., Dierich A., Chambon P., Mark M. (2000). Effect of single and compound knockouts of estrogen receptors alpha (ERalpha) and beta (ERbeta) on mouse reproductive phenotypes. Development.

[34] Critchley H.O., Henderson T.A., Kelly R.W., Scobie G.S., Evans L.R., Groome N.P., Saunders P.T. (2002). Wild-type estrogen receptor (ERbeta1) and the splice variant (ERbetacx/beta2) are both expressed within the human endometrium throughout the normal menstrual cycle. J. Clin. Endocrinol. Metab..

[35] Bulun S.E., Monsavais D., Pavone M.E., Dyson M., Xue Q., Attar E., Tokunaga H., Su E.J. (2012). Role of estrogen receptor-beta in endometriosis. Semin. Reprod. Med..

[36] Hausknecht V., de la Osa E.L., Gurpide E. (1982). In vitro metabolism of C19 steroids in human endometrium. J. Steroid Biochem..

[37] Keski-Rahkonen P., Huhtinen K., Desai R., Harwood D.T., Handelsman D.J., Poutanen M., Auriola S. (2013). LC-MS analysis of estradiol in human serum and endometrial tissue: Comparison of electrospray ionization, atmospheric pressure chemical ionization and atmospheric pressure photoionization. J. Mass Spectrom..

[38] Burger H.G. (2002). Androgen production in women. Fertil. Steril..

[39] Simitsidellis I., Saunders P.T.K., Gibson D.A. (2018). Androgens and endometrium: New insights and new targets. Mol. Cell. Endocrinol..

[40] Gibson D.A., Simitsidellis I., Kelepouri O., Critchley H.O.D., Saunders P.T.K. (2018). Dehydroepiandrosterone enhances decidualization in women of advanced reproductive age. Fertil. Steril..

[41] Rhee H.S., Oh S.H., Ko B.J., Han D.M., Jeon B.H., Park H., Moon H.B., Kim W.S. (2003). Expression of 3β-hydroxysteroid dehydrogenase and P450 side chain cleavage enzyme in the human uterine endometrium. Exp. Mol. Med..

[42] Catalano R.D., Wilson M.R., Boddy S.C., Jabbour H.N. (2011). Comprehensive expression analysis of prostanoid enzymes and receptors in the human endometrium across the menstrual cycle. Mol. Hum. Reprod..

[43] Gibson D.A., Simitsidellis I., Cousins F.L., Critchley H.O., Saunders P.T. (2016). Intracrine Androgens Enhance Decidualization and Modulate Expression of Human Endometrial Receptivity Genes. Sci. Rep..

[44] Ito K., Utsunomiya H., Suzuki T., Saitou S., Akahira J., Okamura K., Yaegashi N., Sasano H. (2006). 17β-hydroxysteroid dehydrogenases in human endometrium and its disorders. Mol. Cell. Endocrinol..

[45] Mustonen M.V., Isomaa V.V., Vaskivuo T., Tapanainen J., Poutanen M.H., Stenback F., Vihko R.K., Vihko P.T. (1998). Human 17β-hydroxysteroid dehydrogenase type 2 messenger ribonucleic acid expression and localization in term placenta and in endometrium during the menstrual cycle. J. Clin. Endocrinol. Metab..

[46] Sivik T., Gunnarsson C., Fornander T., Nordenskjold B., Skoog L., Stal O., Jansson A. (2012). 17β-Hydroxysteroid dehydrogenase type 14 is a predictive marker for tamoxifen response in oestrogen receptor positive breast cancer. PLoS ONE.

[47] Lu M.L., Huang Y.W., Lin S.X. (2002). Purification, reconstitution, and steady-state kinetics of the trans-membrane 17β-hydroxysteroid dehydrogenase 2. J. Biol. Chem..

[48] Gibson D.A., Foster P.A., Simitsidellis I., Critchley H.O.D., Kelepouri O., Collins F., Saunders P.T.K. (2018). Sulfation Pathways: A role for steroid sulphatase in intracrine regulation of endometrial decidualisation. J. Mol. Endocrinol..

[49] Das A., Mantena S.R., Kannan A., Evans D.B., Bagchi M.K., Bagchi I.C. (2009). De novo synthesis of estrogen in pregnant uterus is critical for stromal decidualization and angiogenesis. Proc. Natl. Acad. Sci. USA.

[50] Das A., Li Q., Laws M.J., Kaya H., Bagchi M.K., Bagchi I.C. (2012). Estrogen-induced expression of Fos-related antigen 1 (FRA-1) regulates uterine stromal differentiation and remodeling. J. Biol. Chem..

[51] Dassen H., Punyadeera C., Kamps R., Delvoux B., Van Langendonckt A., Donnez J., Husen B., Thole H., Dunselman G., Groothuis P. (2007). Estrogen metabolizing enzymes in endometrium and endometriosis. Hum. Reprod..

[52] Sinreih M., Knific T., Anko M., Hevir N., Vouk K., Jerin A., Frkovic Grazio S., Rizner T.L. (2017). The Significance of the Sulfatase Pathway for Local Estrogen Formation in Endometrial Cancer. Front. Pharmacol..

[53] Mueller J.W., Gilligan L.C., Idkowiak J., Arlt W., Foster P.A. (2015). The Regulation of Steroid Action by Sulfation and Desulfation. Endocr. Rev..

[54] Rubin G.L., Harrold A.J., Mills J.A., Falany C.N., Coughtrie M.W. (1999). Regulation of sulphotransferase expression in the endometrium during the menstrual cycle, by oral contraceptives and during early pregnancy. Mol. Hum. Reprod..

[55] Greaves E., Temp J., Esnal-Zufiurre A., Mechsner S., Horne A.W., Saunders P.T. (2015). Estradiol Is a Critical Mediator of Macrophage-Nerve Cross Talk in Peritoneal Endometriosis. Am. J. Pathol..

[56] De Leo B., Esnal-Zufiaurre A., Collins F., Critchley H.O.D., Saunders P.T.K. (2017). Immunoprofiling of human uterine mast cells identifies three phenotypes and expression of ERβ and glucocorticoid receptor. F1000Research.

[57] Gibson D.A., Greaves E., Critchley H.O., Saunders P.T. (2015). Estrogen-dependent regulation of human uterine natural killer cells promotes vascular remodelling via secretion of CCL2. Hum. Reprod..

[58] Greaves E., Collins F., Critchley H.O., Saunders P.T. (2013). ERbeta-dependent effects on uterine endothelial cells are cell specific and mediated via Sp1. Hum. Reprod..

[59] Gibson D.A., Simitsidellis I., Saunders P.T. (2016). Regulation of androgen action during establishment of pregnancy. J. Mol. Endocrinol..

[60] Cloke B., Christian M. (2012). The role of androgens and the androgen receptor in cycling endometrium. Mol. Cell. Endocrinol..

[61] Cloke B., Huhtinen K., Fusi L., Kajihara T., Yliheikkila M., Ho K.K., Teklenburg G., Lavery S., Jones M.C., Trew G. (2008). The androgen and progesterone receptors regulate distinct gene networks and cellular functions in decidualizing endometrium. Endocrinology.

[62] Cousins F.L., Kirkwood P.M., Murray A.A., Collins F., Gibson D.A., Saunders P.T. (2016). Androgens regulate scarless repair of the endometrial “wound” in a mouse model of menstruation. FASEB J..

[63] Rothman M.S., Carlson N.E., Xu M., Wang C., Swerdloff R., Lee P., Goh V.H., Ridgway E.C., Wierman M.E. (2011). Reexamination of testosterone, dihydrotestosterone, estradiol and estrone levels across the menstrual cycle and in postmenopausal women measured by liquid chromatography-tandem mass spectrometry. Steroids.

[64] Vercellini P., Vigano P., Somigliana E., Fedele L. (2014). Endometriosis: Pathogenesis and treatment. Nat. Rev. Endocrinol..

[65] Foti P.V., Farina R., Palmucci S., Vizzini I.A.A., Libertini N., Coronella M., Spadola S., Caltabiano R., Iraci M., Basile A. (2018). Endometriosis: Clinical features, MR imaging findings and pathologic correlation. Insights Imaging.

[66] Bulun S.E., Lin Z., Zhao H., Lu M., Amin S., Reierstad S., Chen D. (2009). Regulation of aromatase expression in breast cancer tissue. Ann. N. Y. Acad. Sci..

[67] Brown J., Farquhar C. (2014). Endometriosis: An overview of Cochrane Reviews. Cochrane Database Syst. Rev..

[68] Bulun S.E., Monsivais D., Kakinuma T., Furukawa Y., Bernardi L., Pavone M.E., Dyson M. (2015). Molecular biology of endometriosis: From aromatase to genomic abnormalities. Semin. Reprod. Med..

[69] Attar E., Bulun S.E. (2006). Aromatase and other steroidogenic genes in endometriosis: Translational aspects. Hum. Reprod. Update.

[70] Cheong Y.C., Shelton J.B., Laird S.M., Richmond M., Kudesia G., Li T.C., Ledger W.L. (2002). IL-1, IL-6 and TNF-α concentrations in the peritoneal fluid of women with pelvic adhesions. Hum. Reprod..

[71] Skrzypczak J., Jedrzejczak P., Kasprzak M., Puk E., Kurpisz M. (1995). Inflammatory cytokines in peritoneal fluid of women with endometriosis. Ann. N. Y. Acad. Sci..

[72] Noble L.S., Takayama K., Zeitoun K.M., Putman J.M., Johns D.A., Hinshelwood M.M., Agarwal V.R., Zhao Y., Carr B.R., Bulun S.E. (1997). Prostaglandin E2 stimulates aromatase expression in endometriosis-derived stromal cells. J. Clin. Endocrinol. Metab..

[73] Karck U., Reister F., Schafer W., Zahradnik H.P., Breckwoldt M. (1996). PGE2 and PGF2α release by human peritoneal macrophages in endometriosis. Prostaglandins.

[74] Attar E., Tokunaga H., Imir G., Yilmaz M.B., Redwine D., Putman M., Gurates B., Attar R., Yaegashi N., Hales D.B. (2009). Prostaglandin E2 via steroidogenic factor-1 coordinately regulates transcription of steroidogenic genes necessary for estrogen synthesis in endometriosis. J. Clin. Endocrinol. Metab..

[75] Crosbie E.J., Zwahlen M., Kitchener H.C., Egger M., Renehan A.G. (2010). Body mass index, hormone replacement therapy, and endometrial cancer risk: A meta-analysis. Cancer Epidemiol. Biomarkers Prev..

[76] Sanderson P.A., Critchley H.O., Williams A.R., Arends M.J., Saunders P.T. (2017). New concepts for an old problem: The diagnosis of endometrial hyperplasia. Hum. Reprod. Update.

[77] Rizner T.L., Thalhammer T., Ozvegy-Laczka C. (2017). The Importance of Steroid Uptake and Intracrine Action in Endometrial and Ovarian Cancers. Front. Pharmacol..

[78] Simpson E.R. (2004). Aromatase: Biologic relevance of tissue-specific expression. Semin. Reprod. Med..

[79] Sales K.J., Jabbour H.N. (2003). Cyclooxygenase enzymes and prostaglandins in pathology of the endometrium. Reproduction.

[80] Bulun S.E., Chen D., Lu M., Zhao H., Cheng Y., Demura M., Yilmaz B., Martin R., Utsunomiya H., Thung S. (2007). Aromatase excess in cancers of breast, endometrium and ovary. J. Steroid Biochem. Mol. Biol..

[81] Sasano H., Kaga K., Sato S., Yajima A., Nagura H., Harada N. (1996). Aromatase cytochrome P450 gene expression in endometrial carcinoma. Br. J. Cancer.

[82] Utsunomiya H., Ito K., Suzuki T., Kitamura T., Kaneko C., Nakata T., Niikura H., Okamura K., Yaegashi N., Sasano H. (2004). Steroid sulfatase and estrogen sulfotransferase in human endometrial carcinoma. Clin. Cancer Res..

[83] Utsunomiya H., Suzuki T., Kaneko C., Takeyama J., Nakamura J., Kimura K., Yoshihama M., Harada N., Ito K., Konno R. (2001). The analyses of 17β-hydroxysteroid dehydrogenase isozymes in human endometrial hyperplasia and carcinoma. J. Clin. Endocrinol. Metab..

[84] Cornel K.M., Krakstad C., Delvoux B., Xanthoulea S., Jori B., Bongers M.Y., Konings G.F., Kooreman L.F., Kruitwagen R.F., Salvesen H.B. (2017). High mRNA levels of 17β-hydroxysteroid dehydrogenase type 1 correlate with poor prognosis in endometrial cancer. Mol. Cell. Endocrinol..

[85] Segawa T., Shozu M., Murakami K., Kasai T., Shinohara K., Nomura K., Ohno S., Inoue M. (2005). Aromatase expression in stromal cells of endometrioid endometrial cancer correlates with poor survival. Clin. Cancer Res..

[86] Kamal A.M., Bulmer J.N., DeCruze S.B., Stringfellow H.F., Martin-Hirsch P., Hapangama D.K. (2016). Androgen receptors are acquired by healthy postmenopausal endometrial epithelium and their subsequent loss in endometrial cancer is associated with poor survival. Br. J. Cancer.

[87] Tanaka S., Miki Y., Hashimoto C., Takagi K., Doe Z., Li B., Yaegashi N., Suzuki T., Ito K. (2015). The role of 5α-reductase type 1 associated with intratumoral dihydrotestosterone concentrations in human endometrial carcinoma. Mol. Cell. Endocrinol..

[88] Schiffer L., Arlt W., Storbeck K.H. (2018). Intracrine androgen biosynthesis, metabolism and action revisited. Mol. Cell. Endocrinol..

[89] Dubois V., Laurent M., Boonen S., Vanderschueren D., Claessens F. (2012). Androgens and skeletal muscle: Cellular and molecular action mechanisms underlying the anabolic actions. Cell. Mol. Life Sci..

[90] Dubois V., Laurent M.R., Sinnesael M., Cielen N., Helsen C., Clinckemalie L., Spans L., Gayan-Ramirez G., Deldicque L., Hespel P. (2014). A satellite cell-specific knockout of the androgen receptor reveals myostatin as a direct androgen target in skeletal muscle. FASEB J..

[91] Dalton J.T., Barnette K.G., Bohl C.E., Hancock M.L., Rodriguez D., Dodson S.T., Morton R.A., Steiner M.S. (2011). The selective androgen receptor modulator GTx-024 (enobosarm) improves lean body mass and physical function in healthy elderly men and postmenopausal women: Results of a double-blind, placebo-controlled phase II trial. J. Cachexia Sarcopenia Muscle.

[92] Dalton J.T., Taylor R.P., Mohler M.L., Steiner M.S. (2013). Selective androgen receptor modulators for the prevention and treatment of muscle wasting associated with cancer. Curr. Opin. Support. Palliat. Care.

[93] Labrie F., Archer D.F., Bouchard C., Girard G., Ayotte N., Gallagher J.C., Cusan L., Baron M., Blouin F., Waldbaum A.S. (2015). Prasterone has parallel beneficial effects on the main symptoms of vulvovaginal atrophy: 52-week open-label study. Maturitas.

[94] Martel C., Labrie F., Archer D.F., Ke Y., Gonthier R., Simard J.N., Lavoie L., Vaillancourt M., Montesino M., Balser J. (2016). Serum steroid concentrations remain within normal postmenopausal values in women receiving daily 6.5 mg intravaginal prasterone for 12 weeks. J. Steroid Biochem. Mol. Biol..

[95] Labrie F., Martel C. (2017). A low dose (6.5 mg) of intravaginal DHEA permits a strictly local action while maintaining all serum estrogens or androgens as well as their metabolites within normal values. Horm. Mol. Biol. Clin. Investig..

[96] Caruso S., Cianci S., Vitale S.G., Matarazzo M.G., Amore F.F., Cianci A. (2017). Effects of ultralow topical estriol dose on vaginal health and quality of life in postmenopausal women who underwent surgical treatment for pelvic organ prolapse. Menopause.

[97] Labrie F. (2018). Intracrinology and menopause: The science describing the cell-specific intracellular formation of estrogens and androgens from DHEA and their strictly local action and inactivation in peripheral tissues. Menopause.

[98] Yang S., Fang Z., Suzuki T., Sasano H., Zhou J., Gurates B., Tamura M., Ferrer K., Bulun S. (2002). Regulation of aromatase P450 expression in endometriotic and endometrial stromal cells by CCAAT/enhancer binding proteins (C/EBPs): Decreased C/EBPbeta in endometriosis is associated with overexpression of aromatase. J. Clin. Endocrinol. Metab..

[99] Pavone M.E., Bulun S.E. (2012). Aromatase inhibitors for the treatment of endometriosis. Fertil. Steril..

[100] Colette S., Donnez J. (2011). Are aromatase inhibitors effective in endometriosis treatment?. Expert Opin. Investig. Drugs.

[101] O’Reilly M.W., Kempegowda P., Walsh M., Taylor A.E., Manolopoulos K.N., Allwood J.W., Semple R.K., Hebenstreit D., Dunn W.B., Tomlinson J.W. (2017). AKR1C3-mediated adipose androgen generation drives lipotoxicity in women with polycystic ovary syndrome. J. Clin. Endocrinol. Metab..

[102] Day J.M., Foster P.A., Tutill H.J., Parsons M.F., Newman S.P., Chander S.K., Allan G.M., Lawrence H.R., Vicker N., Potter B.V. (2008). 17β-hydroxysteroid dehydrogenase Type 1, and not Type 12, is a target for endocrine therapy of hormone-dependent breast cancer. Int. J. Cancer.

[103] Delvoux B., D’Hooghe T., Kyama C., Koskimies P., Hermans R.J., Dunselman G.A., Romano A. (2014). Inhibition of type 1 17β-hydroxysteroid dehydrogenase impairs the synthesis of 17β-estradiol in endometriosis lesions. J. Clin. Endocrinol. Metab..

[104] Konings G.F., Cornel K.M., Xanthoulea S., Delvoux B., Skowron M.A., Kooreman L., Koskimies P., Krakstad C., Salvesen H.B., van Kuijk K. (2018). Blocking 17β-hydroxysteroid dehydrogenase type 1 in endometrial cancer: A potential novel endocrine therapeutic approach. J. Pathol..

[105] Rizner T.L. (2016). The Important Roles of Steroid Sulfatase and Sulfotransferases in Gynecological Diseases. Front. Pharmacol..

[106] Piccinato C.A., Neme R.M., Torres N., Sanches L.R., Derogis P., Brudniewski H.F., Rosa E.S.J.C., Ferriani R.A. (2016). Effects of steroid hormone on estrogen sulfotransferase and on steroid sulfatase expression in endometriosis tissue and stromal cells. J. Steroid Biochem. Mol. Biol..

[107] Purohit A., Foster P.A. (2012). Steroid sulfatase inhibitors for estrogen- and androgen-dependent cancers. J. Endocrinol..

[108] Colette S., Defrere S., Lousse J.C., Van Langendonckt A., Gotteland J.P., Loumaye E., Donnez J. (2011). Inhibition of steroid sulfatase decreases endometriosis in an in vivo murine model. Hum. Reprod..

[109] Salah M., Abdelsamie A.S., Frotscher M. (2017). First Dual Inhibitors of Steroid Sulfatase (STS) and 17β-Hydroxysteroid Dehydrogenase Type 1 (17beta-HSD1): Designed Multiple Ligands as Novel Potential Therapeutics for Estrogen-Dependent Diseases. J. Med. Chem..

[110] Potter B.V.L. (2018). Sulfation Pathways: Steroid sulphatase inhibition via aryl sulphamates: Clinical progress, mechanism and future prospects. J. Mol. Endocrinol..

[111] Ferlita A., Battaglia R., Andronico F., Caruso S., Cianci A., Purrello M., Pietro C.D. (2018). Non-Coding RNAs in Endometrial Physiopathology. Int. J. Mol. Sci..

